# Novel circular RNA circ-0002727 regulates miR-144-3p/KIF14 pathway to promote lung adenocarcinoma progression

**DOI:** 10.3389/fcell.2023.1249174

**Published:** 2023-11-14

**Authors:** Yang Li, Xiu Hong, Jingfang Zhai, Ying Liu, Rui Li, Xiuli Wang, Youwei Zhang, Qian Lv

**Affiliations:** ^1^ Department of Central Laboratory, Xuzhou Central Hospital, Xuzhou, China; ^2^ Department of Prenatal Diagnosis Medical Center, Xuzhou Central Hospital, Xuzhou, China; ^3^ Department of Medical Oncology, Xuzhou Central Hospital, Xuzhou, China

**Keywords:** circular RNA, lung adenocarcinoma, competing endogenous RNA, miR-144-3p, KIF14

## Abstract

**Objective:** Circular RNAs (circRNAs) have been shown to participate in various cancers via sponging miRNAs (microRNAs). However, their role in lung adenocarcinoma (LUAD) remains elusive.

**Methods:** The transcriptome data and corresponding clinical information of lung adenocarcinoma samples were extracted from The Cancer Genome Atlas (TCGA) and Gene Expression Omnibus (GEO) database. Differentially expressed circRNAs (DEcircRNAs), differentially expressed miRNAs (DEmiRNAs), and differentially expressed genes (DEgenes) were identified and further used to constructed a circRNA-associated competing endogenous RNA (ceRNA) network. Real-Time qPCR analysis was conducted to examine gene expression at transcriptional level. The regulatory mechanisms of circRNA-miRNA-gene were validated by dual-luciferase reporter array and RNA pull-down assay. Cell growth, migration and invasion were evaluated by CCK-8 assay, colony formation assay and transwell assay, respectively.

**Results:** Based on public microarray data, we systematically constructed a circRNA-associated ceRNA network including 11 DEcircRNAs, 8 DEmiRNAs and 49 DEgenes. Among the ceRNA network, we found that circ-0002727 was a key regulatory and was further confirmed to be upregulated in LUAD cancer cells. Subsequently, we found that silencing of circ-0002727 significantly suppressed the LUAD cell proliferation, migration and invasion *in vitro*. Mechanistically, we showed that circ-0002727 could competitively bind miR-144-3p to enhance the KIF14 expression in LUAD cells. Rescue assays indicated that circ-0002727 could regulate LUAD cell proliferation through modulating miR-144-3p/KIF14 pathway. Besides, KIF14 expression level was positively correlated with TNM stage and metastasis, and patients with high KIF14 expression suffered poor prognosis.

**Conclusion:** Taken together, our study revealed that circ-0002727 could act as a ceRNA to regulate LUAD progression via modulating miR-144-3p/KIF14 pathway, providing a potential therapeutic target for LUAD.

## 1 Introduction

Lung cancer is one of the most prevalent cancers and the leading cause of cancer-related death worldwide (Siegel et al., 2021). As the major histological subtype, lung adenocarcinoma (LUAD) accounts for ∼40% of all cases and is increasing year by year. Despite improvements of diagnostic and therapeutic approaches, the 5-year overall survival rate of LUAD remains very poor due to the tumor recurrence, metastasis and drug resistance (Hirsch et al., 2017). Thus, it is urgently needed to explore the underlying pathogenesis and develop novel therapeutic targets for LUAD.

With the development of sequencing technology and bioinformatics methods, increasing circular RNAs (circRNAs) were identified and attracted great research interest as a type of regulatory RNAs. CircRNAs are characterized by the covalent closed-loop structure lack of 3′ and 5′ ends that are produced by precursor mRNA (pre-mRNA). Recent studies imply that circRNAs are abundant, conserved stable and transcribed in a tissue-specific manner (Salzman, 2016). With the accumulating knowledge of characteristics and actions of circRNAs, it has been confirmed that circRNAs play crucial roles in human diseases (Zhang et al., 2018; Altesha et al., 2019; Jin et al., 2020), especially in cancer (Chen and Shan, 2021; Kristensen et al., 2022; Xue et al., 2022). For example, Wang et al. (2021) reported that circURI1 exhibited the significantly higher expression in gastric cancer compared with corresponding non-tumor adjacent specimens and inhibitory effects on tumor metastasis. Zhi et al. (2021) found that circRNA-102049 was highly expressed in primary colorectal cancer with liver metastasis and closely correlated with the prognosis of patients with colorectal cancer. A growing number of researches have strongly illustrated that circRNAs were closely involved in tumorigenesis and progression. Hence, exploring the underlying regulatory mechanism of circRNAs may benefit for tumor therapy.

Recent researches have widely reported that circRNAs functioning as microRNA sponges through the competing endogenous RNA (ceRNA) network to regulate gene expression at the transcriptional or post-transcriptional level in various types of cancer (Liu et al., 2019; Yuan et al., 2021; Zhang et al., 2021). For instance, Huang G. et al. (2020) reported that circular RNA hsa-circRNA-104348 could act as a ceRNA to promote hepatocellular carcinoma progression by targeting miR-187-3p/RTKN2 axis and activating Wnt/β-catenin pathway. In addition, another circRNA, named circRNA-0025202, has been confirmed to regulate tamoxifen sensitivity and tumor progression via acting as a miRNA sponge for miR-182-5p and further regulating the expression and activity of FOXO3a in breast cancer (Sang et al., 2019). For LUAD, several dysregulated circRNAs have been identified that were involved in tumorigenesis and progression (Zhou et al., 2019; Huang Q. et al., 2020; Zhang X. et al., 2022). CircRNA-002178 could enhance PDL1 expression via sponging miR-34 to induce T-cell exhaustion in LUAD (Wang et al., 2020). Circ-0018414 acts as a tumor inhibitor in LUAD by sponging miR-6807-3p and further upregulates DKK1 expression (Yao et al., 2021). Although many researches already presented underlying regulatory mechanisms of circRNA, understanding of circRNA function remains insufficient and requires comprehensive exploration for LUAD.

In this study, based on the microarray data extracted from Gene Expression Omnibus (GEO) and The Cancer Genome Atlas (TCGA), we comprehensively investigated the expression characteristics of circRNA, microRNA and gene and further constructed a circRNA-associated ceRNA network to detect the aberrant key circRNAs and potential regulatory mechanism in LUAD. Then, a specific circRNA named circ-0002727 is initially identified, which is significantly upregulated in LUAD tissues and cells. Based on the previously constructed ceRNA network, we aimed to demonstrate that circ-0002727 could act as a ceRNA by sponging miR-144-3p and further upregulates KIF14 expression. Our findings revealed the underlying regulatory mechanisms of oncogenic circ-0002727, which might provide new therapeutic target for LUAD treatment.

## 2 Materials and methods

### 2.1 Patients’ characteristics

Two circRNA expression profiles (GSE101586 and GSE101684) were obtained from the GEO (https://www.ncbi.nlm.nih.gov/geo/) database, including 5 and 4 pairs of LUAD tissues and adjacent normal tissues respectively. Raw data was processed by background correction and normalization using the “affy” package of R/Bioconductor. The expression data of mRNA (513 LUAD tissues and 59 normal tissues) and miRNA (513 LUAD tissues and 46 normal tissues) were extracted from TCGA (https://portal.gdc.cancer.gov/). The level 3 normalized count values were extracted as gene expression measurements. Clinical information of 513 LUAD patients was collected. Due to the missing survival time, age, and tumor stage, 466 LUAD patients were finally retained in our study as described in Table 1.

**TABLE 1:** Clinical information of TCGA-LUAD cohort.

	TCGA-LUAD
Sample	
Normal	—
Tumor	466
Mean age (years; range)	65 (33–88)
Gender	
Male	213
Female	253
Stage	
I	254
II	110
III	77
IV	25
Lymphatic node metastasis	
No	307
Yes	156
Unknown	3
Distant metastasis	
No	310
Yes	24
Unknown	132
Status	
Alive	347
Dead	119
Platform	Illumina HiSeqV2

### 2.2 Constructing the ceRNA network

Differentially expressed circRNAs (DEcircRNAs) were identified between LUAD and adjacent normal tissues using Student’s *t*-test with *p* < 0.05. DEcircRNAs overlapped between GSE101586 and GSE101684 were selected. The differentially expressed miRNAs (DEmiRNAs) and differentially expressed genes (DEgenes) were identified by the “edgeR” package with the threshold of FDR<0.05 and |log2FC|>2. The ceRNA network was constructed based on the interactions among DEcircRNAs, DEmiRNAs and DEmRNAs. The StarBase database (https://starbase.sysu.edu.cn/) was used to predict interactions between DEcircRNAs and DEmiRNAs using miRanda program. The interactions between DEmiRNAs and DEgenes were predicted by Targetscan and were further calculated the expression correlation using Pearson Correlation Coefficient. Only the interactions with significant negative correlation were retained. After removing the nodes that could not form a circRNA-miRNA-gene axis, a ceRNA network was constructed and further visualized by Cytoscape software (www.cytoscape.org). Functional annotations of DEgenes within ceRNA network were performed using The Database for Annotation, Visualization and Integrated Discovery (DAVID, https://david.ncifcrf.gov/). Three categories, including biological processes, molecular function and cellular components, were involved in Gene oncology (GO). Kyoto Encyclopedia of Genes and Genomes (KEGG, https://www.kegg.jp/) was used to carry out the pathway enrichment. The criterion for significant enrichment was *p* < 0.05.

### 2.3 Cell culture and vector transfection

Three human LUAD cells (SPC-A1、HCC827、PC-9 and A549) and one human normal bronchial epithelial cell line (16HBE) were purchased from American Type Culture Collection (ATCC, United States) and were tested negative for *mycoplasma* contamination. All the cells were cultured in Roswell Park Memorial Institute 1,640 (RPMI-1640) medium with 10% FBS under the standard conditions with 5% CO_2_ atmosphere with 37°C. For cell transfection, short interfering RNA (siRNA) target circ-0002727 or negative control (NC), miR-144-3p mimic, inhibitor, and their control plasmids (NC mimic, NC inhibitor) were purchased from Sangon Biotech (Shanghai, China). Transfection (miRNA mimics, inhibitors and siRNAs) was performed using the Lipofectamine 3,000 kit (Invitrogen) according to the manufacturer’s instructions. The siRNA sequences are listed in Supplementary Table S1.

### 2.4 RNA extraction and real-time qPCR

Total RNAs were extracted from cultured cells using Trizol kit (Invitrogen, United States) according to manufacturer’s protocol. RNA was reverse-transcribed using HiScript II Q RT SuperMixfor qPCR (+gDNA wiper) (Vazyme, Nanjing, China). AceQ qPCR SYBR Green Master Mix (Vazyme, Nanjing, China) was used for qRT-PCR. GAPDH and U6 applied as internal references for circRNA/mRNA and miRNA, respectively. The relative expression levels were determined by the 2^−ΔΔCT^ or 2^−ΔΔCT^ method. The primer sequences were listed in Table 2.

**TABLE 2:** Primer sequences for Real-Time qPCR.

Genes	Forward (5′-3′)	Reverse (5′-3′)
Circ-0002727	TGA​GAG​CAC​ATG​GGA​TGG​TA	CCC​AGG​CAG​TTC​TTC​TGA​AT
KIF14	CCC​TCA​CCC​ACA​GTA​GCC​GA	GGG​GTA​AGG​GGC​ATG​TCT​GC
CEP55	CGA​CCG​TCA​ACA​TGT​GCA​GC	GCG​ATG​CTC​AGT​GGC​TGG​AT
PRR11	AAT​GTG​CCT​GCC​TGC​GTT​CT	AGG​AGG​TGG​CAG​AGG​TGG​AG
GAPDH	GCG​GGG​CTC​TCC​AGA​ACA​TC	TCC​ACC​ACT​GAC​ACG​TTG​GC
miR-144-3p	TAC​AGT​ATA​GAT​GAT​GTA​CT	TGGTGTCGTGGAGTCG
miR-185-5p	TGG​AGA​GAA​AGG​CAG​TTC​CTG​A	TGGTGTCGTGGAGTCG
U6	CTCGCTTCGGCAGCACA	AAC​GCT​TCA​CGA​ATT​TGC​GT

### 2.5 CCK-8 assay

Cell Counting Kit-8 assay (BBI, E606335) was performed to measure cell viability according to manufacturer’s instructions. For the cell proliferation assay, 3 × 103 cells were seeded in 100 μL of complete culture media in 96-well plates for various time periods (24, 48, 72 h). After that, the optical density (OD) values were measured in the wavelength of 450 nm to quantify the proliferation abilities of cells.

### 2.6 Clone formation assay

Different groups of transfected LUAD cells were seeded into six‐well plates. Subsequently, the plates were placed in standard culture media for 10 days. After 10 days, colonies were stained with Crystal Violet and counted. Clone formation rate = (number of clones/number of inoculated cells) ×100%. Camera (Olympus, Tokyo, Japan) was used for photographing colonies.

### 2.7 Transwell assay

Transwell 24-well Plate Cell Culture Chamber (Corning, 3,422, Shanghai, China) was used to perform invasion assay. Briefly, the cells were placed in the upper chamber with serum-free RPMI 1640 and the lower chamber was filled with culture medium with 10% fetal bovine serum (FBS). The cells were fixed by 4% paraformaldehyde and stained with 0.1% crystal violet (Sangon, Shanghai, China). The cells were observed and photographed under a 100-fold microscope, and 3 fields were selected to count the cells to reflect cell mobility.

### 2.8 RNA pull down

RNAs were labeled with biotin using Pierce RNA 3ʹ End Desthiobiotinylation Kit (Thermo Fisher Scientific, Waltham, MA, United States) according to the manufacturer’s protocols. Biotin-labeled wild-type and mutant miR-144-3p RNA probes were synthesized and transfected into cells. After incubation with cell lysates and magnetic beads for 6 h, the precipitated RNAs were eluted and extracted by TRizol method, followed by qRT-PCR examination to detect circ-0002727 and GAPDH.

### 2.9 Dual-luciferase reporter assay

Two LUAD cell lines (A549 and PC-9) were cultured and seeded into 96-well plates at a density of 5 × 103 cells per well for 24 h before transfection. Then the pmirGLO reporter vector (Promega) carrying wild-type (WT) or mutant type (MUT) circ-0002727 and KIF14 was transfected into cells combination with miR-144-3p mimics or NC mimics using Lipofectamine 2000 (Invitrogen, United States). After 48 h, the luciferase values were normalized to the corresponding Renila luciferase values, and then the fold changes were calculated.

### 2.10 Statistical analysis

All results were expressed as mean ± SD. Student’s *t*-test or Kruskal–Wallis test was utilized to calculate significant difference. Survival curves were estimated using the Kaplan-Meier method and were compared using the log-rank test. The significance was defined as a *p*-value of <0.05. All statistical analysis was performed using GraphPad Prism 8 and R3.4.0.

## 3 Results

### 3.1 Construction of the circRNA-associated ceRNA network in LUAD

**FIGURE 1:**
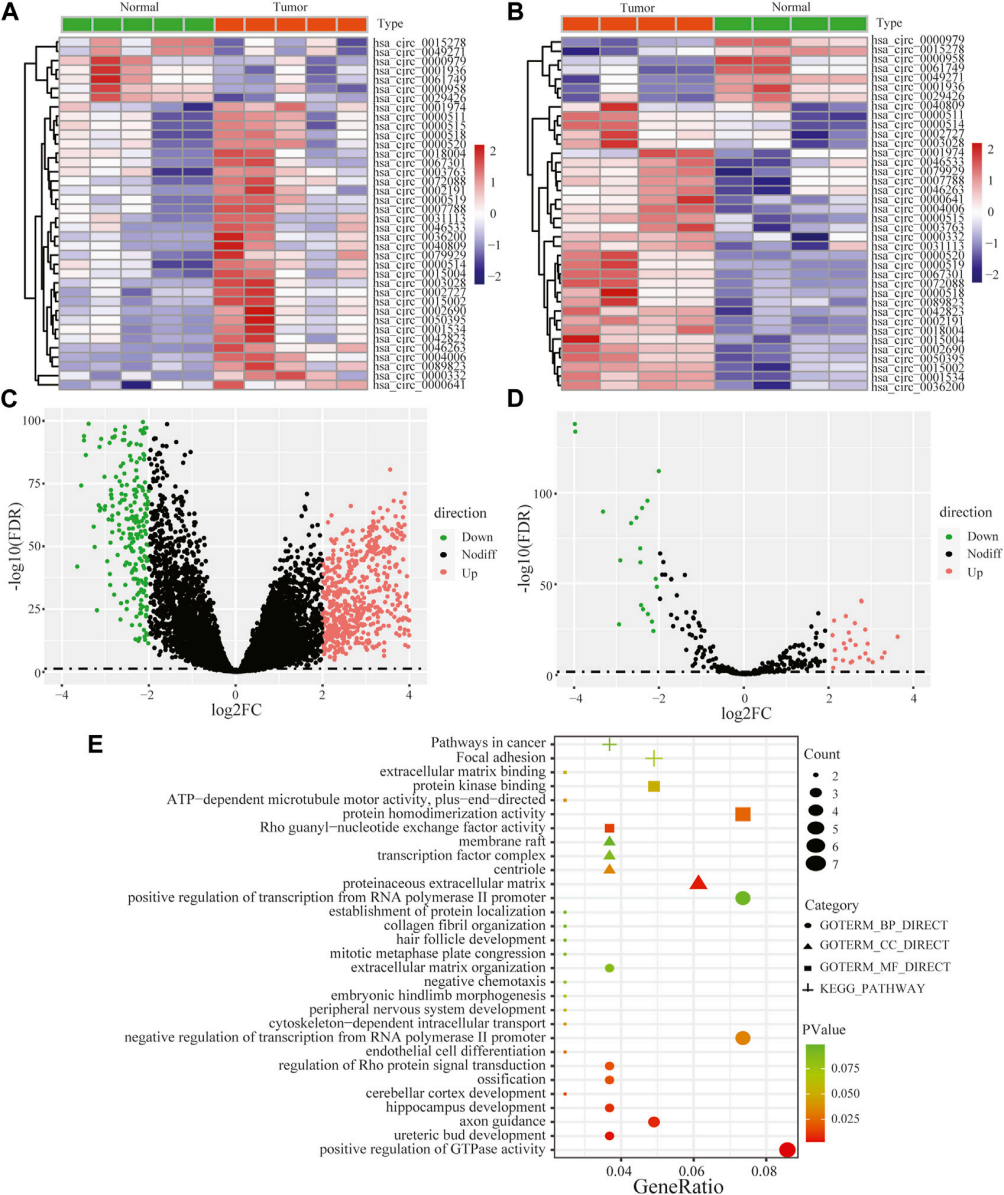
Heat maps of differentially expressed circular RNAs based on GSE101586 **(A)** and GSE101684 **(B)**, volcano plots of differentially expressed genes **(C)** and differentially expressed miRNA **(D)** and functional enrichment of differentially expressed genes within ceRNA network **(E)**.

**FIGURE 2:**
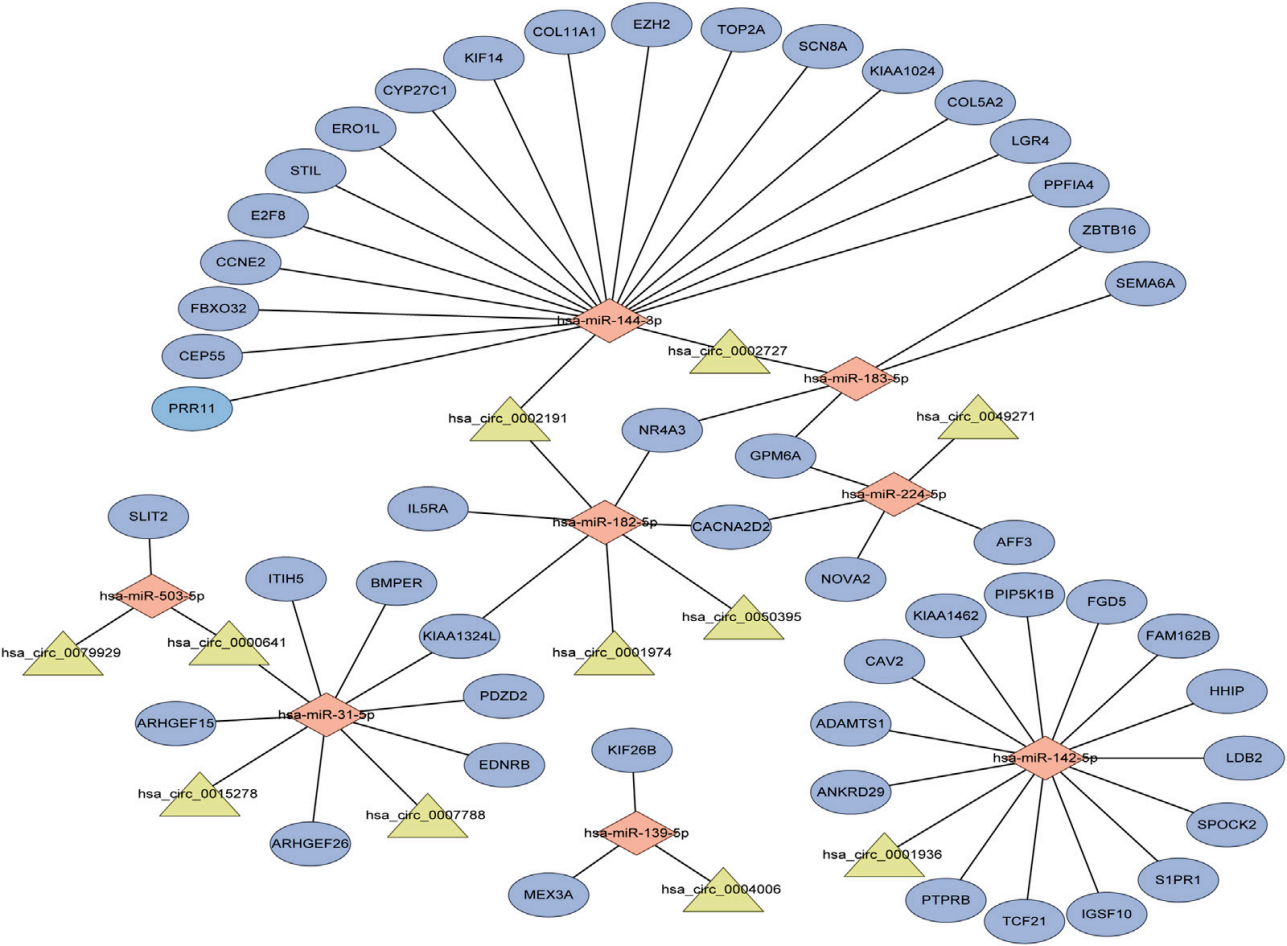
The circRNA-associated competing endogenous network in lung adenocarcinoma. Triangles represent circRNAs, diamonds represent miRNAs, ellipses represent genes, and black lines represent circRNA-miRNA-gene interactions.

Performing differential expression analysis between LUAD and adjacent normal tissues, we identified 38 DEcircRNAs (including 31 upregulated circRNAs and 7 downregulated circRNAs) overlapped between GSE101586 and GSE101684 (Figures 1A, B). Moreover, 50 DEmiRNAs (including 34 upregulated miRNAs and 16 downregulated miRNAs, Figure 1D) and 960 DEgenes (including 653 upregulated genes and 307 downregulated genes, Figure 1C) were identified based on TCGA-LUAD dataset, respectively. Using the online StarBase database, 39 DEcircRNA-DEmiRNA pairs were predicted. Moreover, 53 DEmiRNA-DEgene pairs with significant negative expression correlation were identified based on the Targetscan prediction tool and Pearson Correlation Coefficient. Combining the DEcircRNA-DEmiRNA pairs with DEmiRNA-DEgene pairs, we finally constructed a circRNA-miRNA-gene ceRNA network, including 11 DEcircRNAs, 8 DEmiRNAs and 49 DEgenes (Figure 2). Functional enrichment analysis showed that the ceRNA network involved in many cancer-related pathways, such as positive regulation of GTPase activity, Regulation of Rho protein signal transduction, endothelial cell differentiation, and regulation of transcription from RNA polymerase II promotion (Figure 1E).

### 3.2 Characterization of circ-0002727 in LUAD tissues and cells

**FIGURE 3:**
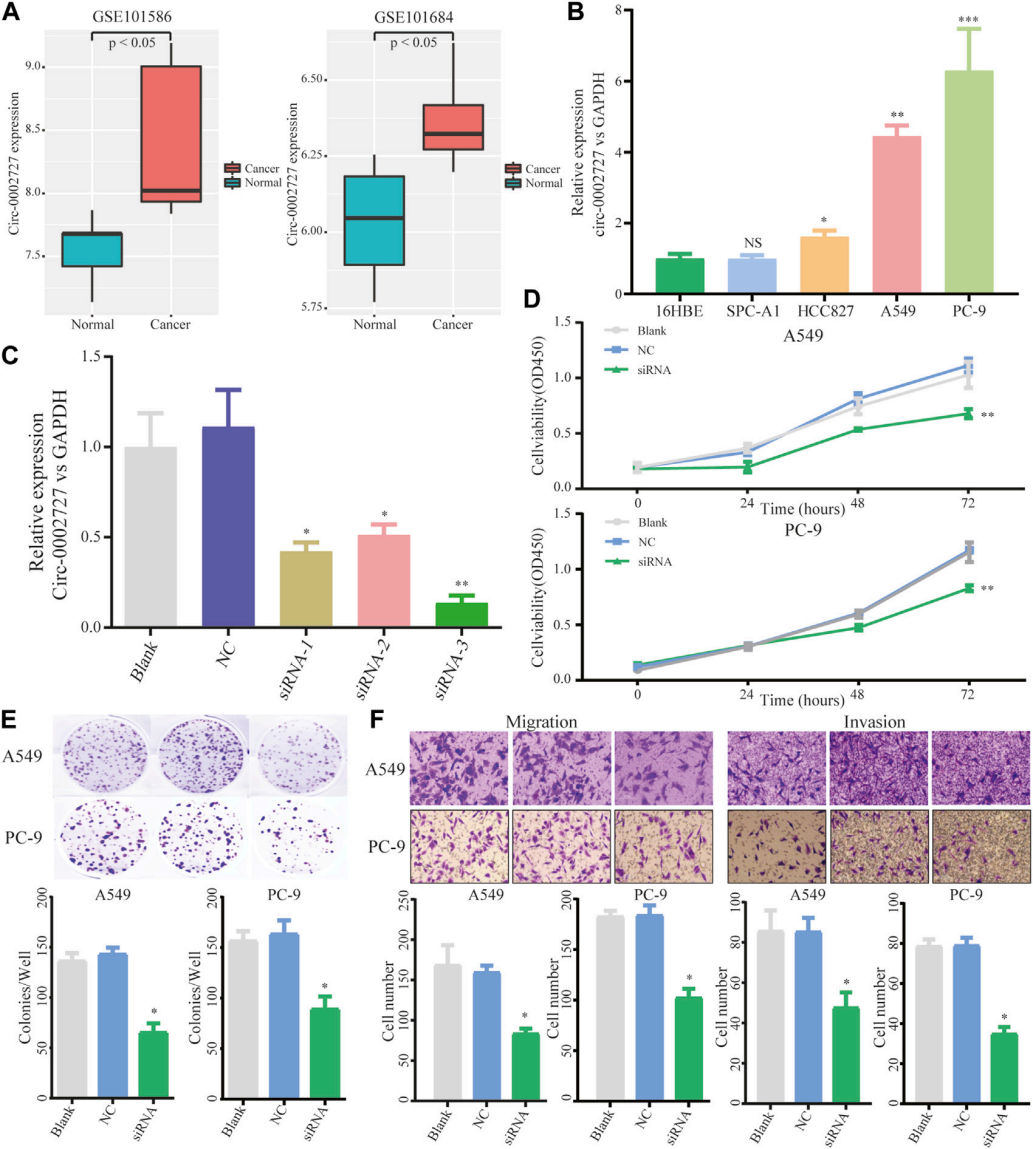
CircRNA circ-0002727 was highly expressed in LUAD tissues and cells. **(A)** The expression of circ-0002727 was upregulated in LUAD tissues; **(B)** The expression of circ-0002727 was detected in LUAD cell lines (A549, PC-9, HCC827, and SPC-A1) and normal human bronchial epithelial cell line (16HBE) by qRT-PCR; **(C)** Relative expression of circ-0002727 in LUAD cells transfected with three kinds of siRNAs from the CircInteractome database; **(D–F)** The effect of circ-0002727 on LUAD cell proliferation, migration and invasion was determined by CCK-8 **(D)**, colony formation **(E)**, and Transwell assay **(F)** respectively. Data were expressed as mean ± SD. *p < 0.05, **p < 0.01, NS means no statistical significance.

We found that circ-0002727 was a key regulatory within the ceRNA network. In addition, circ-0002727 was significantly upregulated in LUAD tissues compared with adjacent normal tissues based on the microarray data of circRNA expression collected from the GEO database (Figure 3A). Thus, we selected circ-0002727 as our protagonist in the current study. We further observed the expression of circ-0002727 in four kinds of LUAD cell line (A549, PC-9, HCC827, and SPC-A1) and normal human bronchial epithelial cell line (16HBE). The results showed that the expression of circ-0002727 was significantly higher in three LUAD cancer cell lines (A549, PC-9, and HCC827) than that in 16HBE cell line, but no significant difference in SPC-A1 cell line (Figure 3B). Based on these results, we selected A549 and PC-9 cell lines to perform further cell function experiments in order to obtain full expression spectrum of circ-0002727 in LUAD cells. To test whether circ-0002727 was functional in LUAD, we generated 3 siRNAs from the CircInteractome database for gene abrogation experiments (Supplementary Table S1). The results showed that all siRNA-1, siRNA-2, and siRNA-3 treatment resulted in less than 50% of the residual circ-0002727 level seen with blank and siCtrl (Figure 3C). An approximately 90% reduction of circ-0002727 in siRNA-3 treatment cells was observed. Then, we performed short interfering RNAs (siRNA-3 treatment) in further experiments. Cell proliferation assay revealed that silencing of circ-0002727 significantly suppressed cell growth (Figure 3D). Colony formation assay also confirmed the significant reduction of colonies under silencing of circ-0002727 (Figure 3E). We further performed transwell assays using A549 and PC-9 cells to evaluate the association between circ-0002727 expression and metastases. The results showed that the circ-0002727 knockdown remarkably repressed the migration and invasion of LUAD cells (Figure 3F).

### 3.3 Circ-0002727 served as a sponge of miR-144-3p

**FIGURE 4:**
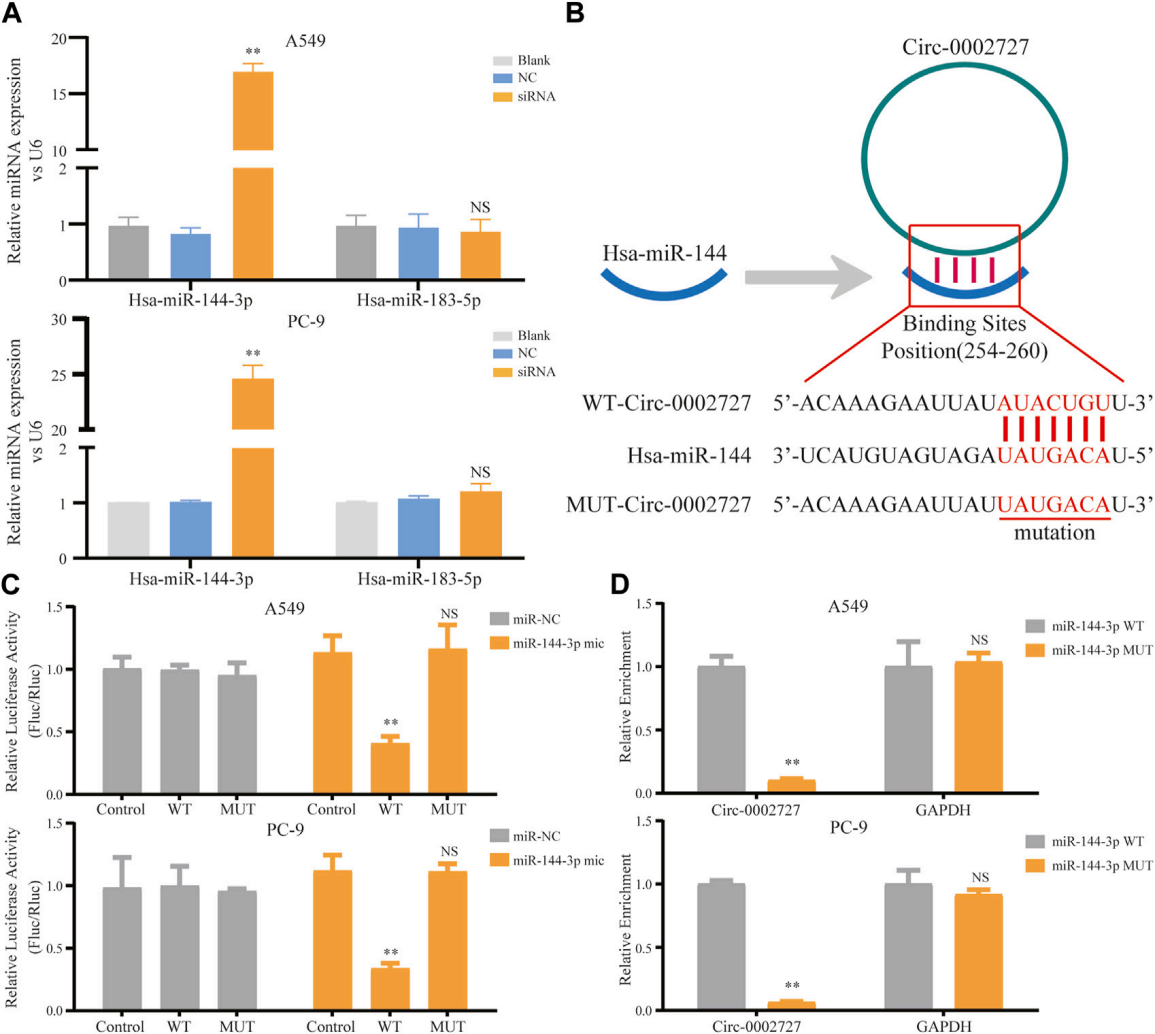
CircRNA circ-0002727 inhibited miRNA miR-144-3p expression in LUAD cells by acting as a RNA sponge. **(A)** Relative expression of predicted targets (miR-144-3p and miR-183-5p) in LUAD cell lines (A549 and PC-9) transfected with si-circ-0002727; **(B)** The binding sites of circ-0002727 and miR-144-3p were predicted by using the online starBase software; **(C)** Dual-luciferase reporter assay was performed to validate the binding sites of circ-0002727 and miR-144-3p; **(D)** MiR-144-3p enriched in the pull-down production with the circ-0002727 probe in A549 and PC-9 cell lines. *p < 0.05, **p < 0.01, NS means no statistical significance.

Based on the previously constructed ceRNA network, we predicted the possible miRNA target of circ-0002727 using the online StarBase software. As shown in ceRNA network, two miRNAs including miR-144-3p and mir-183-5p were the potential target of circ-0002727. We then detected the relative expression of these two miRNAs determined in circ-0002727 knockdown system. The results showed that the level of miR-144-3p was significantly upregulated after transfected with siRNA (*p* < 0.05), while there was no change in expression of miR-183-5p in both A549 and PC-9 LUAD cell lines (Figure 4A). Then, we hypothesized that miR-144-3p could be a target of circ-0002727 (Figure 4B). We further performed the dual-luciferase reporter array and RNA pull-down assay experiments to confirm that miR-144-3p was the downstream target of circ-0002727. The dual-luciferase reporter array results showed that miR-144-3p mimic significantly decreased the luciferase activity co-transfected with Wt-circ-0002727 instead of Mut-circ-0002727 in both A549 and PC-9 LUAD cell lines (Figure 4C). Consistently, the RNA pull-down assay validated that circRNA-0002727 was specific enriched in the miR-144-3p WT group compared with miR-144-3p MUT group (Figure 4D). These results confirmed that circ-0002727 functioned as RNA sponges to inhibit miR-144-3p in LUAD cells.

### 3.4 KIF14 was a direct target of miR-144-3p

**FIGURE 5:**
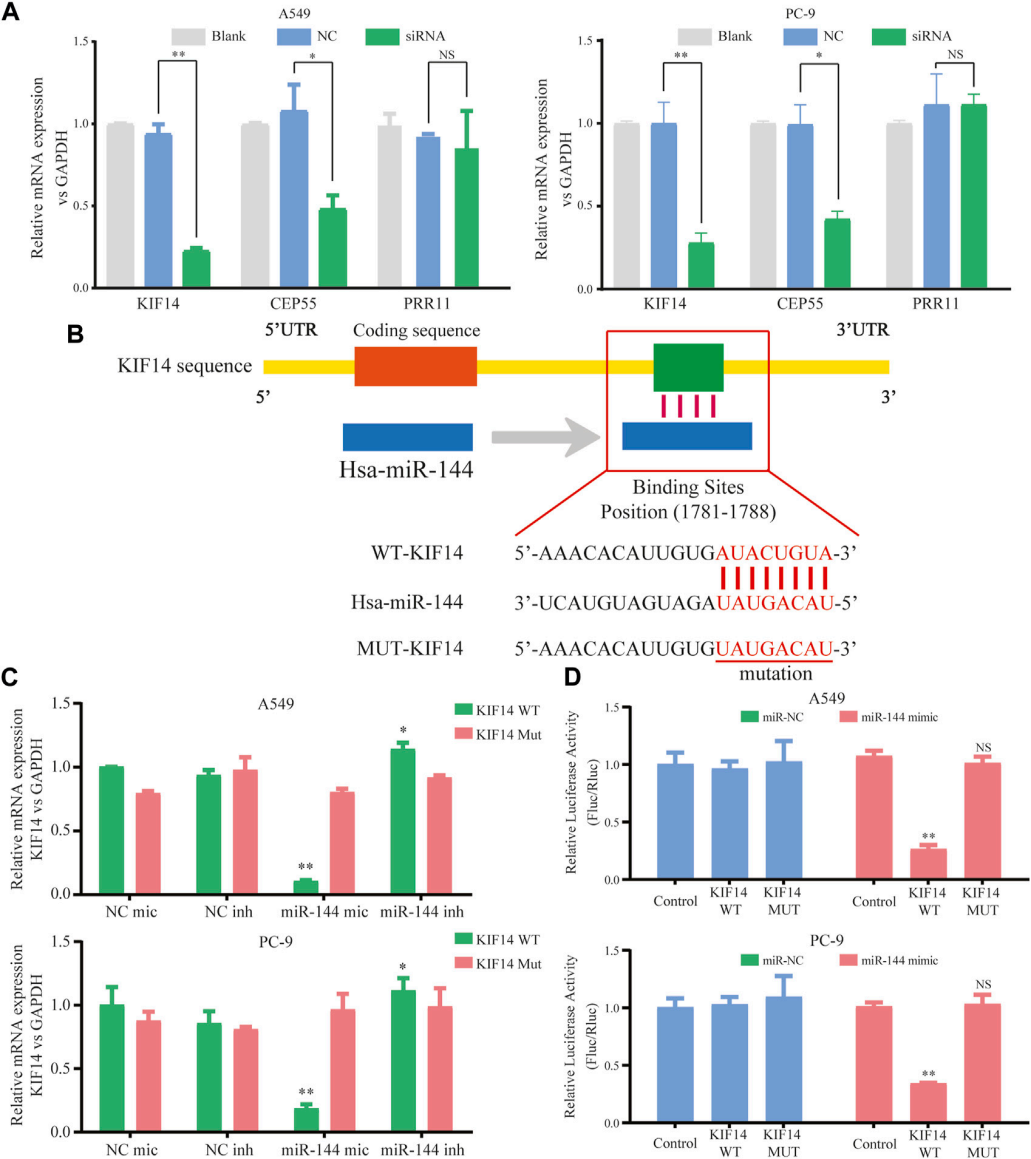
KIF14 could be negatively regulated by miR-144-3p in LUAD cells. **(A)** Relative expression of predicted targets (KIF14, CEP55, and PRR11) in LUAD cell lines (A549 and PC-9) transfected with si-circ-0002727; **(B)** The binding sites of KIF14 and miR-144-3p were predicted by using Targetscan database; **(C)** KIF14 mRNA was negatively regulated by miR-144-3p in both A549 and PC-9 cell lines; **(D)** Dual-luciferase reporter assay was performed to validate the binding sites of KIF14 and miR-144-3p. *p < 0.05, **p < 0.01, NS means no statistical significance.

Within the ceRNA network, 12 genes were predicted as potential targets of the miR-144-3p. Here, we selected three genes (including KIF14, CEP55, and PRR11) with the highest expression correlation between miR-144-3p based on the expression data from TCGA-LUAD (Supplementary Table S2). We then detected the relative expression of three genes based on the circ-0002727 knockdown system. The results showed that the expression level of KIF14 was most significantly downregulated after transfected with siRNA (*p* < 0.05), while there was no change in expression of PRR11 in both A549 and PC-9 LUAD cell lines (Figure 5A). Using the online StarBase software, we predicted the binding sites of miR-144-3p and the 3′ untranslated region (3′-UTR) of KIF14 mRNA (Figure 5B). Thus, we selected KIF14 as the optimal target gene and performed a series of experiments to better understand the underlying regulatory mechanisms. The expression of KIF14 mRNA was detected in miR-144-3p mimic and inhibitor system using A549 and PC-9 LUAD cell lines, respectively. Results showed that expression of KIF14 mRNA was negatively regulated by miR-144-3p (Figure 5C). Additionally, the relationship between miR-144-3p and KIF14 was explored by the dual-luciferase reporter array. The results showed that miR-144-3p mimics inhibited the activity of WT-KIF14 reporter in both A549 and PC-9 LUAD cell lines, indicating KIF14 was a direct target of miR-144-3p (Figure 5D).

### 3.5 Circ-0002727 regulated LUAD progression, metastasis and prognosis by modulating miR-144-3p/KIF14 pathway

**FIGURE 6:**
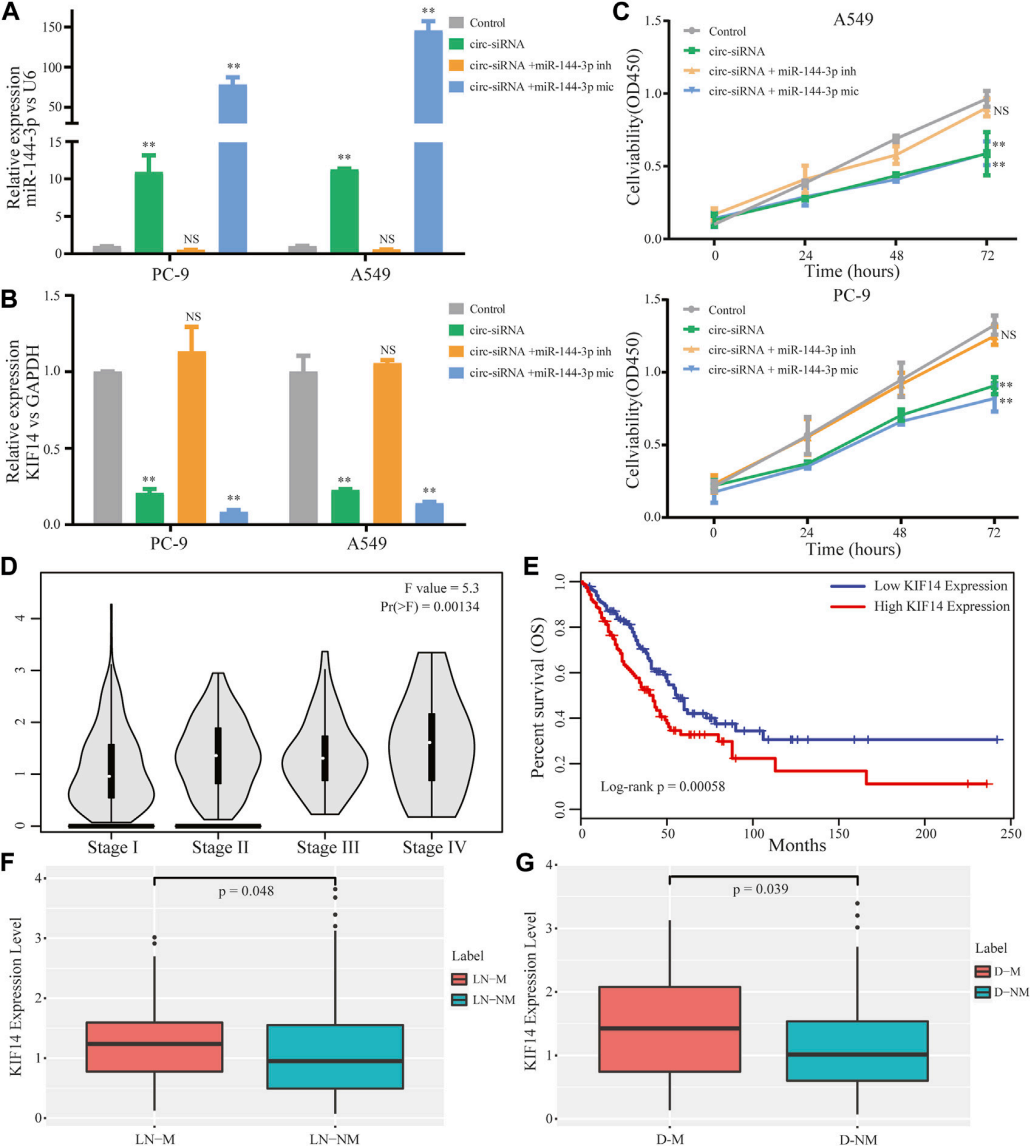
CircRNA circ-0002727 regulated KIF14 in LUAD cells by targeting miR-144-3p and could have effect on LUAD progression and prognosis. A549 and PC-9 cell lines were co-transfected with circ-siRNA or NC and miR-144-3p inhibitor or inhibitor. **(A,B)** Relative expression of KIF14 and miR-144-3p were detected by qRT-PCR in A549 and PC-9 cell lines; **(C)** Cell proliferation abilities were determined by CCK-8 assay; **(D)** Expression difference of KIF14 among different stage; **(E)** Kaplan-Meier survival analysis was performed to analyze the correlations of KIF14 with LUAD patients prognosis; **(F)** Expression difference of KIF14 between lymphatic node metastasis (LN-M) and nonmetastasis (LN-NM) samples; **(G)** Expression difference of KIF14 between distant metastasis (D-M) and nonmetastasis (D-NM) samples. *p < 0.05, **p < 0.01, NS means no statistical significance.

The above results enlightened us to speculate that circ-0002727 could regulate KIF14 expression in LUAD cells by targeting miR-144-3p. To validate this hypothesis, Sh-circRNA or sh-NC and miR-144-3p inhibitor or mimic were co-transfected in A549 and PC-9 LUAD cell lines respectively. As expected, the miR-144-3p remarkably increased when the circRNA-0002727 was knockdowned by the specific siRNAs (Figure 6A), while this increase could be abolished when co-transfected with miR-144-3p inhibitor. Similarly, KIF14 was significantly downregulated in LUAD cells treated with specific circRNA-0002727 siRNAs, while such decrease was counteracted when co-transfected with miR-144-3p inhibitor (Figure 6B). In addition, these phenomena were even more pronounced when co-transfected with miR-144-3p mimic (Figures 6A, B). CCK-8 assay results showed that cell viability was greatly suppressed by knockdown of circ-0002727, while this effect could be compromised when co-transfected with miR-144-3p inhibitor in both A549 and PC-9 LUAD cell lines (Figure 6C). Taken together, our results confirmed that circ-0002727 could enhance KIF14 expression via absorbing miR-144-3p in LUAD cells. To further explore the effect of effector gene KIF14 on LUAD progression, we analyzed the association between KIF14 expression and clinical stage, lymph node metastasis, distant metastasis as well as prognosis. We found that KIF14 was significantly differentially expressed among stage I-IV (Figure 6D). Moreover, KIF14 were significantly upregulated in patients with lymphatic node metastasis and in patients with distant metastasis, respectively (Figures 6E, F). Furthermore, patients with high KIF14 expression had significantly shorter OS than patients with low KIF14 expression (Figure 6G). These results indicated that circ-0002727 could regulate the KIF14 expression by modulating miR-144-3p, thereby participating in LUAD progression, metastasis and prognosis.

## 4 Discussion

With the development of high-throughput sequencing technology and bioinformatics methods, an increasing number of circRNAs have been identified acting as tumor promoters or suppressors (Su et al., 2019; Cao et al., 2020; Liu et al., 2020). The expression profiling provides a prerequisite for exploring their characteristics and functions. Numerous evidences have demonstrated that the dysregulation of circRNA could be closely related to the LUAD progression (He et al., 2020; Zhang T. et al., 2022; Wang et al., 2022). However, the expression pattern and biological function of circRNAs in LUAD are still not fully understood. In this study, we constructed a circRNA-associated ceRNA network by comprehensively analyzing the expression profiles of circRNA, miRNA and gene and identified a key circRNA named circ-0002727 that could promote the LUAD progression by modulating miR-144-3p/KIF14 axis.

CircRNAs have been demonstrated to participate in various pathologic processes, including apoptosis, proliferation and invasion. For example, circular RNA circBCBM1 is involved in breast cancer brain metastasis via circBCBM1/miR-125a/BRD4 axis (Fu et al., 2021). CircRNA has-circ-0001946 promotes cell growth in lung adenocarcinoma by regulating miR-135a-5p/SIRT1 axis and activating Wnt/β-catenin signaling pathway (Yao et al., 2019). In our study, we identified circ-0002727 acting as a significantly upregulated circRNA in LUAD tissues and cells based on the LUAD tissue-based microarray data and PCR data of LUAD cell lines. Loss-of-function experiments demonstrated that downregulated circ-0002727 could inhibit proliferation, migration, and invasion of LUAD cells, indicating that circ-0002727 indeed plays a functional regulatory role in LUAD progression.

Recent studies have shown that circRNAs could regulate gene expression through multiple mechanisms, such as acting as miRNA sponges, promoting gene transcription, regulating alternative splicing, inhibiting RNA maturation (Ashwal-Fluss et al., 2014; Guo et al., 2014; Li et al., 2015; Kristensen et al., 2018). To explore the regulatory mechanism of circ-0002727, we used the bioinformatics tools (miRanda) to predict the potential target of circ-0002727, and finally confirmed that miR-144-3p was the bio-target of circ-0002727 by the dual-luciferase reporter array and RNA pull-down assay, indicating that circ-0002727 could competitively bind miR-144-3p to regulate the expression of downstream target genes. Many researches have demonstrated that miR-144-3p is closely involved in the tumor progression and chemotherapy response (Wu et al., 2019; Sun et al., 2020; Lu et al., 2021; Cui et al., 2022). Jiang et al. (2020) found that miR-144-3p was significantly downregulated in lung cancer cell lines and could effectively suppressed TGF-β1-induced lung cancer cell invasion and adhesion by regulating the Src-Akt-Erk pathway. Another study found that miR-144-3p expression was downregulated and could inhibit the Nrf2 pathway during the cisplatin resistance process in lung cancer cells (Yin et al., 2018). These results indicated that circ-0002727 might bind to miR-144-3p to affect tumor progression and chemotherapy response.

KIF14 can act as a chromokinesin via binding to microtubules and chromatin during the bipolar spindle formation. Many studies have reported that KIF14 could promote tumorigenesis and progression by acting on many signaling pathways (Yang et al., 2019; Zhang J. et al., 2022). Zhang J. et al. (2022) found that KIF14 is an oncogene in cervical cancer, and knocking down KIF14 causes cell cycle arrest by inhibiting p27 degradation, thus affecting cell viability, proliferation, and migration. Similarly, silencing of KIF14 repressed tumor cell growth by interfering with cytokinesis and the p27Kip1 ubiquitination pathway in hepatocellular carcinoma (Xu et al., 2014). Corson et al. (2007) found that KIF14 expression is independently prognostic for disease-free survival in lung cancer and knockdown decreases tumorigenicity *in vitro*, showing that it is a clinically relevant oncogene and an exciting therapeutic target. In our study, we found that circ-0002727 shared the common MRE of miR-144-3p with the KIF14 and the KIF14 expression was significantly downregulated in LUAD cells transfected with siRNA. Through bioinformatics analysis and a series of experiments, we also confirmed that miR-144-3p could regulate KIF14 expression via binding with 3′-UTR of KIF14 at post-transcriptional level. Furthermore, we demonstrated that circ-0002727 could serve as a ceRNA for miR-144-3p to further regulate the expression of KIF14 in LUAD cells, elucidating the regulatory mechanism of circ-0002727 involved in LUAD progression and providing the potential therapeutic target.

In conclusion, our study for the first time constructed a LUAD circRNA-associated ceRNA network and demonstrated that circ-0002727 promoted LUAD malignant behaviors via modulating miR-144-3p/KIF14 axis, which provided a novel potential regulatory mechanism involved in LUAD progression and helpful guidance for LUAD therapy as promising therapeutic targets in the near future.

## Data Availability

The original contributions presented in the study are included in the article/Supplementary Material, further inquiries can be directed to the corresponding authors.
