# Supplementary material for: Novel circular RNA circ-0002727 regulates miR-144-3p/KIF14 pathway to promote lung adenocarcinoma progression
Source: Front Cell Dev Biol. 2023 Nov 14;11:1249174. doi: 10.3389/fcell.2023.1249174 (PMC10686231; doi:10.3389/fcell.2023.1249174)
Supplement: Supplementary file 1 [file Table1.DOCX]

Supplementary Material

Novel circular RNA circ-0002727 regulates miR-144-3p/KIF14 pathway to promote lung adenocarcinoma progression

Yang Li^1†^, Xiu Hong^1†^, Jingfang Zhai^2†^, Ying Liu^1^, Rui Li^1^, Xiuli Wang^1^, Youwei Zhang^3*^, Qian Lv^1*^

^1^Department of Central laboratory, Xuzhou Central hospital, Xuzhou, China

^2^Department of Prenatal Diagnosis Medical Center, Xuzhou Central Hospital, Xuzhou, China

^3^Department of Medical Oncology, Xuzhou Central hospital, Xuzhou, China

**†: These authors contributed equally to this work and share first authorship.**

*** Correspondence:**Qian Lv; Email: [lvqian0625@126.com](mailto:lvqian0625@126.com)

Youwei Zhang; Email: zhangyw@njmu.edu.cn

Table S1: The siRNA sequences generated from the CircInteractome database

| **Gene** | **Sense (5’-3’)** | **Antisense (3’-5’)** |
| --- | --- | --- |
| circ-0002727 siRNA-1 | AGCACCAUCAGUCACCCACAAUU | UUUCGUGGUAGUCAGUGGGUGUU |
| circ-0002727 siRNA-2 | GCACCAUCAGUCACCCACAAAUU | UUCGUGGUAGUCAGUGGGUGUUU |
| circ-0002727 siRNA-3 | CAUCAGUCACCCACAAAAGCUUU | UUGUAGUCAGUGGGUGUUUUCGA |

Table S2: Expression correlation between miR-144-3p and predicted targets

| miRNAs | Genes | P.value | Estimate |
| --- | --- | --- | --- |
| hsa-mir-144-3p | KIF14 | 0.00014 | -0.16575 |
| hsa-mir-144-3p | CEP55 | 0.00014 | -0.16459 |
| hsa-mir-144-3p | PRR11 | 0.00015 | -0.16442 |
| hsa-mir-144-3p | EZH2 | 0.00015 | -0.16432 |
| hsa-mir-144-3p | KIAA1024 | 0.00018 | -0.16203 |
| hsa-mir-144-3p | PPFIA4 | 0.00031 | -0.15618 |
| hsa-mir-144-3p | CYP27C1 | 0.00049 | -0.15094 |
| hsa-mir-144-3p | COL5A2 | 0.00109 | -0.14159 |
| hsa-mir-144-3p | E2F8 | 0.00117 | -0.14080 |
| hsa-mir-144-3p | TOP2A | 0.00167 | -0.13633 |
| hsa-mir-144-3p | STIL | 0.00187 | -0.13496 |
| hsa-mir-144-3p | SCN8A | 0.00246 | -0.13140 |
| hsa-mir-144-3p | ERO1L | 0.00268 | -0.13026 |
| hsa-mir-144-3p | COL11A1 | 0.00568 | -0.12009 |
| hsa-mir-144-3p | FBXO32 | 0.00602 | -0.11928 |
| hsa-mir-144-3p | LGR4 | 0.00850 | -0.11431 |
| hsa-mir-144-3p | CCNE2 | 0.02126 | -0.10013 |
